# Corrigendum

**DOI:** 10.1002/ehf2.13259

**Published:** 2021-02-04

In the paper by Dong *et al*.,^1^ there was a mistake in the Statistical Analysis under Method section. The sentence ‘Hospitalization for HF, pulmonary diseases, non‐HF CV diseases, and other reasons were treated as time‐dependent covariates, for the status of hospitalization changed over time’ should not have been included. Hospitalization was not included as time‐varying variables in this model. Corresponding results presented in Tables 3 and 4 were yielded from Cox models without time‐varying variables. Similarly, in the Abstract, the regression model was mistakenly described as ‘time‐varying Cox proportional hazards model’. The word ‘time‐varying’ should not have been used.

The authors apologize for the mistake and the misleading information.

## References

[1] Dong B , He X , Xue R , Chen Y , Zhao J , Zhu W , Liang W , Wu Z , Wu D , Huang H , Zhou Y , Dong Y , Liu C . Clinical implication of pulmonary hospitalization in heart failure with preserved ejection fraction: from the TOPCAT. ESC Heart Fail 2020; 7: 3801–3809. 10.1002/ehf2.12966 PMC7754907 32964677

